# A high proportion of caseous necrosis, abscess, and granulation tissue formation in spinal tuberculosis

**DOI:** 10.3389/fmicb.2023.1230572

**Published:** 2023-08-14

**Authors:** Runrui Wu, Shanshan Li, Yadong Liu, Hong Zhang, Dongxu Liu, Yuejiao Liu, Wen Chen, Fenghua Wang

**Affiliations:** Department of Pathology, The 8th Medical Center, Chinese PLA General Hospital, Beijing, China

**Keywords:** spinal tuberculosis, pathological feature, drug resistance, gene, caseous necrosis

## Abstract

The special blood circulation, anatomy, and tissue structure of the spine may lead to significant differences in pathological features and drug resistance between spinal tuberculosis and pulmonary tuberculosis. Here, we collected 168 spinal tuberculosis cases and 207 pulmonary tuberculosis cases, and compared their clinical and pathological features as well as drug resistance. From the anatomical location, the highest incidence was of lumbar tuberculosis, followed by thoracic tuberculosis. PET-CT scans showed increased FDG uptake in the diseased vertebrae, discernible peripheral soft tissue shadow, visible internal capsular shadow, and an abnormal increase in FDG uptake. MRI showed infectious lesions in the diseased vertebral body, formation of paravertebral and bilateral psoas muscle abscess, and edema of surrounding soft tissues. As with control tuberculosis, the typical pathological features of spinal tuberculosis were chronic granulomatous inflammation with caseous necrosis. The incidence of granulomas was not statistically different between the groups. However, the proportions of caseous necrosis, acute inflammation, abscess, exudation, and granulation tissue formation in the spinal tuberculosis group were all significantly increased relative to the control tuberculosis group. Compared to the control tuberculosis group, the incidences of resistance to rifampicin (RFP) + isoniazid (INH) + streptomycin (STR) and INH + ethambutol (EMB) were lower in the spinal tuberculosis group, while the incidences of resistance to RFP + INH + EMB and RFP + EMB were higher. Moreover, we also found some differences in drug-resistance gene mutations. In conclusion, there are noticeable differences between spinal *Mycobacterium tuberculosis* and pulmonary tuberculosis in pathological characteristics, drug resistance, and drug resistance gene mutations.

## 1. Introduction

Tuberculosis (TB), a leading cause of mortality worldwide, affects an estimated 10 million individuals annually (Furin et al., 2019). With the emergence of drug-resistant tuberculosis strains and the increase in the HIV infection rate, the number of tuberculosis cases is increasing steadily, and the incidence of osteoarticular tuberculosis is also increasing significantly (Diedrich et al., 2016). As the most severe and prevalent form of bone and joint tuberculosis, spinal tuberculosis, constitutes approximately 50% of these cases (Ali et al., 2019). All segments of the spine may be affected, with the lumbar spine most commonly affected, followed by the thoracic spine (Liu et al., 2019). Cervical and sacral tuberculosis is relatively rare. The initial symptoms, such as mild pain, stiffness, and loss of muscle strength, are typically nonspecific, often leading to misdiagnoses (Liu et al., 2019). The disease can progress to cause postural abnormalities, spinal deformities, and even paralysis (Jain et al., 2018), severely compromising the patient’s quality of life without management (Ali et al., 2019). This suggested a vital role of prompt diagnosis and personalized treatment for improving the prognosis of spinal tuberculosis.

The current diagnosis of tuberculosis involves the evaluation of the patient’s medical history, clinical symptoms, physical signs, laboratory tests, and imaging tests. Magnetic Resonance Imaging (MRI) serves as a sensitive modality for the early detection of spinal tuberculosis (Moorthy and Prabhu, 2002) and provide detailed information on early vertebral inflammation and minor swelling of paravertebral soft tissue (Moorthy and Prabhu, 2002; Ali et al., 2019). Moreover, it can aid in the diagnosis of neurological dysfunction and assessing spinal cord compression (Ali et al., 2019). In addition, histopathological examination, encompassing H&E staining, acid-fast staining, and molecular testing, is regarded as the gold standard for the diagnosis of spinal tuberculosis. The typical pathological change of tuberculosis is chronic granulomatous inflammation, with caseous necrosis (Li et al., 2020). However, the spine’s relatively limited blood circulation and discharge pathway for necrotic substances may lead to a divergence in the pathology of spinal tuberculosis compared to lung tuberculosis. Li. et al. found that the most common pathologic characteristics of spinal tuberculosis were caseous necrosis, multinuclear giant cells, and granulomatous inflammation (Li et al., 2020). Limited studies revealed the differences.

In the management of spinal tuberculosis, it is crucial that antituberculosis drugs are administered adhering to the principles of early initiation, consistency, complete course treatment, combination therapy, and appropriate dosing. In patients without severe vertebral bone damage and instability, anti-tuberculous drugs alone can promote recovery (Suárez et al., 2019). However, for patients who have severe bone destruction, spinal instability, and spinal nerve compression, surgery is necessary, and antituberculosis drugs are used to keep *Mycobacterium tuberculosis* in a resting state (Kumar et al., 2022). Given these factors, personalized selection of antituberculosis drugs is crucial (Rajasekaran and Khandelwal, 2013; Bhosale et al., 2020). Molecular biology techniques and drug sensitivity tests are currently the main means of detecting antituberculosis drug resistance. However, vertebral puncture can easily damage the spinal cord or important blood vessels and nerves, and the risk is high, especially if tuberculosis has destroyed the vertebral body or surrounding supporting tissue. In this study, the pathological features and drug resistance of spinal tuberculosis were examined, to characterize differences between spinal tuberculosis and pulmonary tuberculosis to inform a more individualized approach to drug regimen selection.

## 2. Materials and methods

### 2.1. Participant selection

This study incorporated patients admitted to the 8th Medical Centre of Chinese PLA General Hospital from January 2016 to December 2020. The inclusion criteria were: age over 18 years, spinal surgery or puncture for pathological examination, clinically and pathologically diagnosed *Mycobacterium tuberculosis* infection, and no prior antituberculosis drug treatment before diagnosis. Exclusion criteria encompassed malignancies, severe fungal and bacterial infections, hematologic malignancies, severe trauma, and surgical history. One hundred sixty eight patients with spinal tuberculosis were included and 207 patients with pulmonary tuberculosis were as the control group. This study was approved by the Ethics Committee of the Chinese PLA General Hospital and was performed in accordance with the principles of the Declaration of Helsinki.

### 2.2. Methods

#### 2.2.1. Imaging examination

In this study, 18F-FDG positron emission tomography (PET/CT) and MRI were used to evaluate spinal tuberculosis, and PET/CT and CT were used to evaluate pulmonary tuberculosis (Vorster et al., 2014). PET/CT can evaluate the spinal morphology and extent of involvement, as well as detect other lesions throughout the body (Gao et al., 2014). In addition, PET/CT can provide glucose metabolism characteristics of spinal tuberculosis and evaluate lesion activity, which is of great significance for early diagnosis, differential diagnosis and curative effect evaluation of spinal tuberculosis (Altini et al., 2020). MRI has a higher imaging resolution for soft tissue than CT and X-ray (Kubihal et al., 2022). MRI can improve the sensitivity and specificity of the diagnosis of spinal tuberculosis and help to evaluate the involvement of the lesion.

#### 2.2.2. H&E staining

All tissue samples underwent fixation in 4% paraformaldehyde, paraffin embedding post gradient dehydration, followed by sectioning (3 μm thickness) and mounting on slides. After baking at 72°C for 30 min, slides underwent dewaxing using xylene for two 10-min intervals, sequentially washed with 100, 90, and 80% gradient anhydrous ethanol for 5 min each time and stained with hematoxylin dye for 30 s. Subsequent steps involved differentiation with 10% hydrochloric acid, blue reversion with ammonia, eosin dye application for 5 s, and dehydration with gradient anhydrous ethanol, transparency with xylene, and sealing with neutral resin. Observations of basic histological features from H&E-stained sections were made under a microscope. Representative tissue sections were chosen for subsequent acid-fast staining and gene detection.

#### 2.2.3. Acid-fast staining

Sections (3 μm thickness) were cut from each sample, adhered to slides, and baked at 72°C for 30 min. The dewaxing process was carried out twice with xylene for 10 min, followed by a 5-min wash with 100, 90, and 80% gradient anhydrous ethanol. The sections were then stained with 2–3 drops of carbolic red dye solution for 2 h and decolorized with 1% hydrochloric alcohol until a light pink hue was achieved. Hematoxylin dye was applied for 30 s, followed by differentiation with 10% hydrochloric acid, and blue reversion with ammonia water. Gradient anhydrous ethanol dehydration, xylene transparency, and neutral resin sealing finalized the process.

#### 2.2.4. Identification of mycobacteria

**Figure 1:**
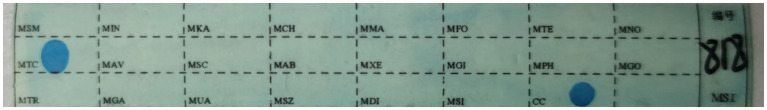
The sequence of the detection site on the membrane strip.

From each patient tissue specimen, 8–10 sections of 5-10 μm thickness were cut and placed in 1.5 mL centrifuge tubes for dewaxing, lysis, digestion, and DNA extraction. PCR tubes from the genetic test kit (Yaneng Biotechnology Co., Ltd., Shenzhen, China) were utilized to identify Mycobacterial species, and 4 μL of sample DNA was added. Following DNA amplification, the membrane strips and amplification products were placed in tubes with 5–6 mL of solution A and heated in a boiling bath for 10 min. They were then hybridized at 59°C for 1.5 h. Post-hybridization, the membrane strips were washed and incubated for 30 min in solution A containing POD enzyme. The reaction was terminated with purified or deionized water after the membrane strips were placed in the chromogenic solution for 10 min. The detection sites were indicated by blue spots on the membrane strips, and each experiment incorporated both positive and negative controls (Zhang et al., 2021; Li et al., 2022; Figure 1).

#### 2.2.5. Mycobacterial drug resistance mutation gene detection

PCR tubes from the Mycobacterial drug resistance mutation gene detection kit (Yaneng Biotechnology Co., Ltd., Shenzhen, China) were used to add 4 μL of sample DNA. After DNA amplification at specific intervals and temperatures, the membrane strips and amplification products were placed in tubes with 5–6 mL of AT solution, heated in a boiling water bath for 10 min, and then hybridized at 59°C for 1.5 h. After washing and incubation, the strips were placed in a chromogenic solution for 10 min, and the reaction was stopped with purified or deionized water. Blue spots on the membrane strips indicated detected sites, with both positive and negative controls performed in each experiment.

### 2.3. Drug resistance types definition

Monoresistance refers to resistance to only one antituberculosis drug (Van Rie et al., 2000). Polyresistance refers to resistance to more than one antituberculosis drug but does not include resistance to RFP and INH (Mizukoshi et al., 2021). Multidrug resistance refers to resistance to at least RFP and INH simultaneously (Van Rie et al., 2000). Drug resistance to any drug refers to resistance to any one or more antituberculosis drugs (Van Rie et al., 2000).

### 2.4. Statistical methods

SPSS25.0 software was used for the statistical analysis. The counting data were described as “case (strain)” and “rate (%).” The chi-square test or Fisher’s exact probability test was used to compare drug resistance rates between groups, and *p* < 0.05 was considered statistically significant.

## 3. Results

### 3.1. Clinical data

**Figure 2:**
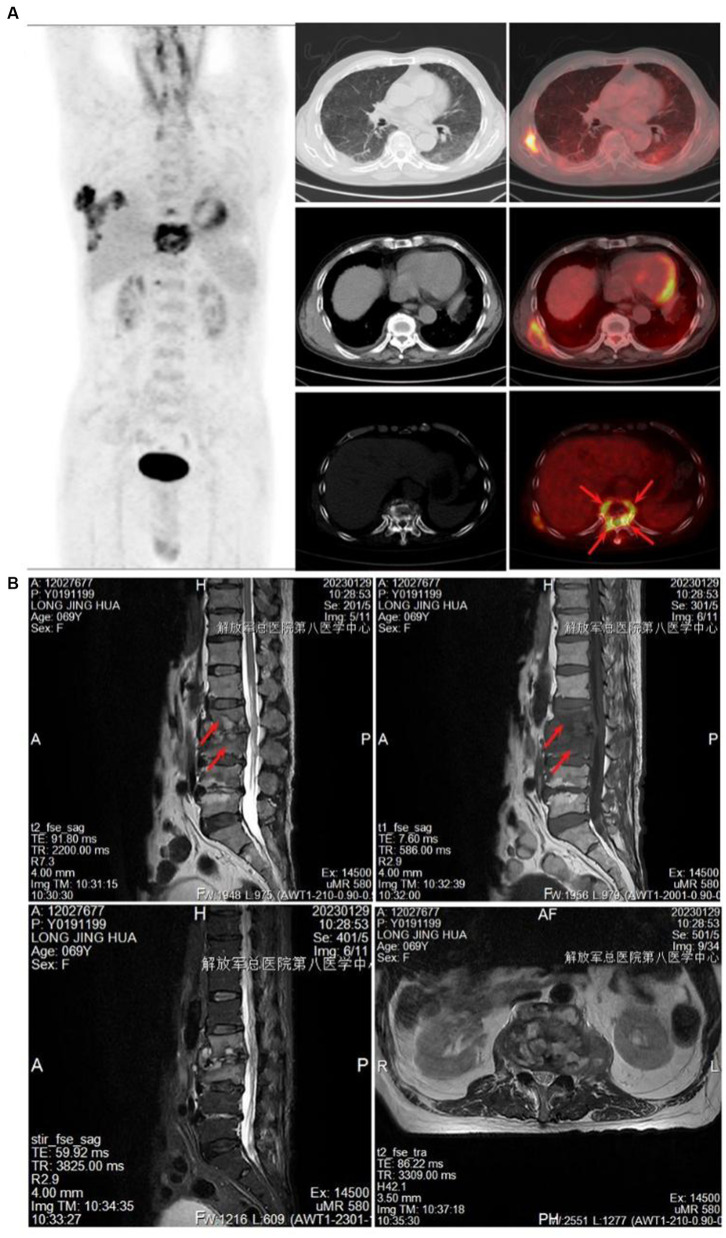
Imaging examination. **(A)** PET-CT showed increased FDG uptake in the diseased vertebrae. **(B)** MRI showed infectious lesions in the diseased vertebral body, the formation of paraspinal and bilateral psoas muscle abscesses, and the edema of the surrounding soft tissues.

The control group consisted of 207 pulmonary tuberculosis patients, including 117 males and 90 females, with a mean age of 43.86 years. The spinal tuberculosis group included 168 patients, with 92 males and 76 females, and a mean age of 43.48 years. The highest incidence was of lumbar tuberculosis (48.81%), followed by thoracic tuberculosis (38.10%). PET-CT scans showed increased FDG uptake in the diseased vertebrae, discernible peripheral soft tissue shadow, visible internal capsular shadow, and an abnormal increase in FDG uptake (Figure 2A). MRI showed infectious lesions in the diseased vertebrae, paraspinal and bilateral lumbar muscle abscess formation, and peripheral soft tissue edema (Figure 2B). PET-CT and CT results of control tuberculosis group were shown in Supplementary Figure S1.

### 3.2. Pathological features

**Figure 3:**
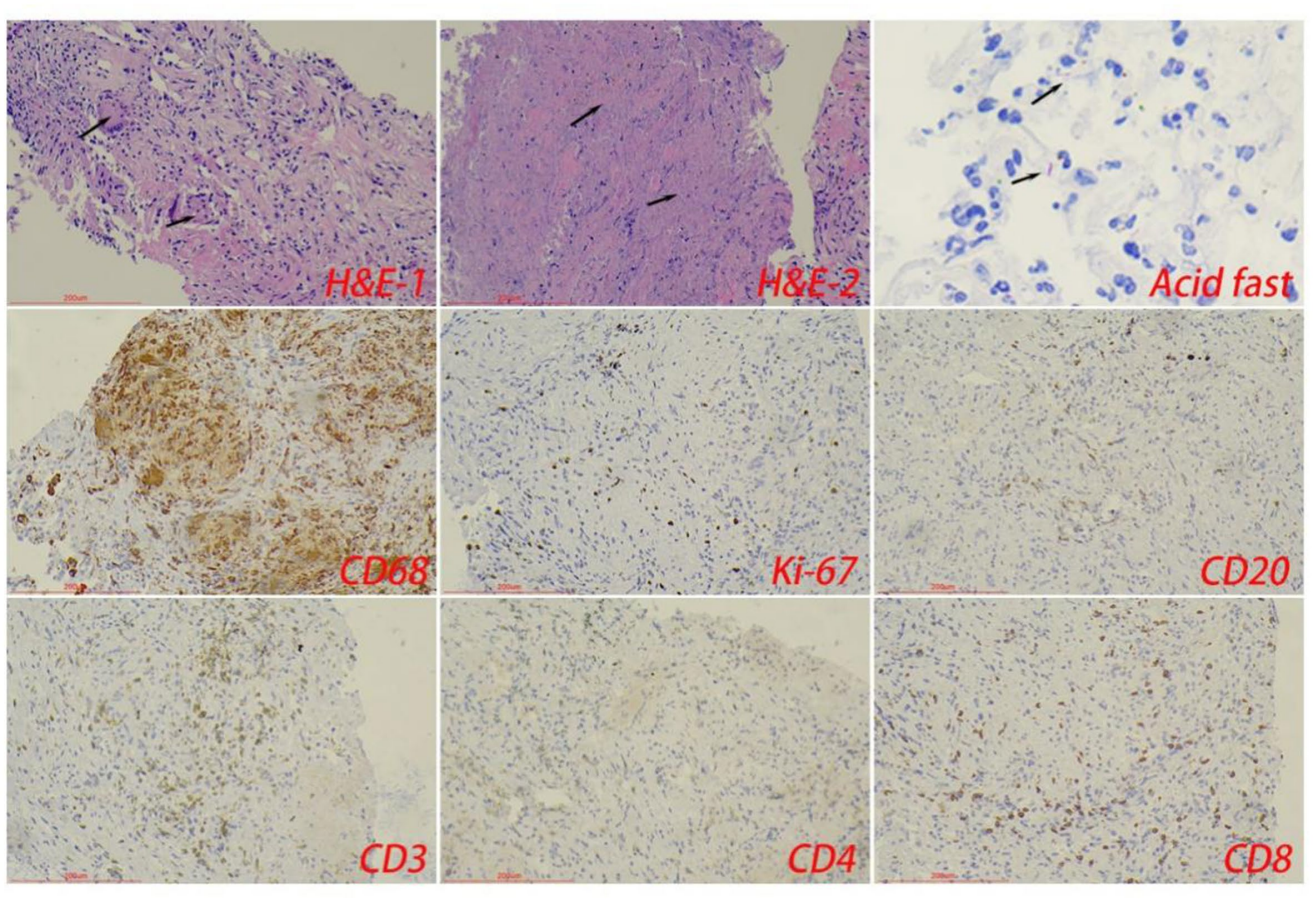
The typical pathological manifestations of pulmonary tuberculosis were chronic granulomatous inflammation (H&E-1) with caseous necrosis (H&E-2). Acid-fast staining showed *Mycobacterium tuberculosis*. Immunohistochemical staining was used to label granuloma (CD68^+^) and inflammatory cell (CD20^+^B cells, CD3^+^T cells, CD4^+^T cells, and CD8^+^T cells) infiltration. Scale bar: 200 μm; Image magnification: H&E (200×), acid-fast staining (400×), immunohistochemical staining (200×).

**Figure 4:**
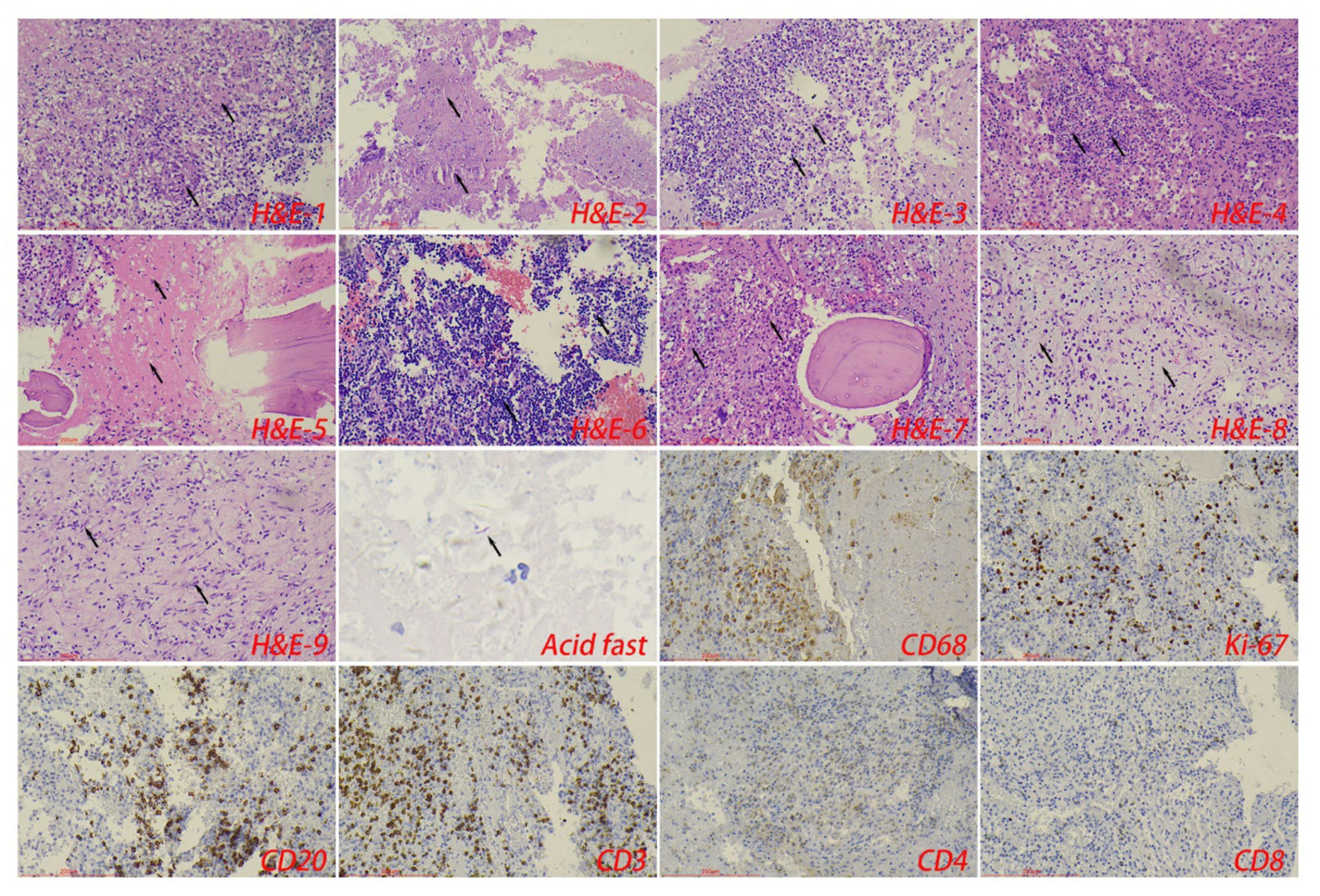
The typical pathological manifestations of spinal tuberculosis were chronic granulomatous inflammation (H&E-1) with caseous necrosis (H&E-2). H&E staining showed abscess (H&E-3), inflammatory necrosis (H&E-4), exudation (H&E-5), acute inflammation (H&E-6), and granulation tissue formation (H&E-7), mucous degeneration (H&E-8), and fibrous tissue hyperplasia (H&E-9). Caseous necrosis is a special type of coagulated necrosis, which is characterized by unstructured granular red staining substance. Inflammatory necrosis is tissue necrosis caused by inflammation, and a large number of acute and chronic inflammatory cells can be observed under the microscope. Acid-fast staining showed *Mycobacterium tuberculosis*. Immunohistochemical staining was used to label granuloma (CD68^+^) and inflammatory cell (CD20^+^B cells, CD3^+^T cells, CD4^+^T cells, and CD8^+^T cells) infiltration. Scale bar: 200 μm; Image magnification: H&E (200×), acid-fast staining (400×), immunohistochemical staining (200×).

**Figure 5:**
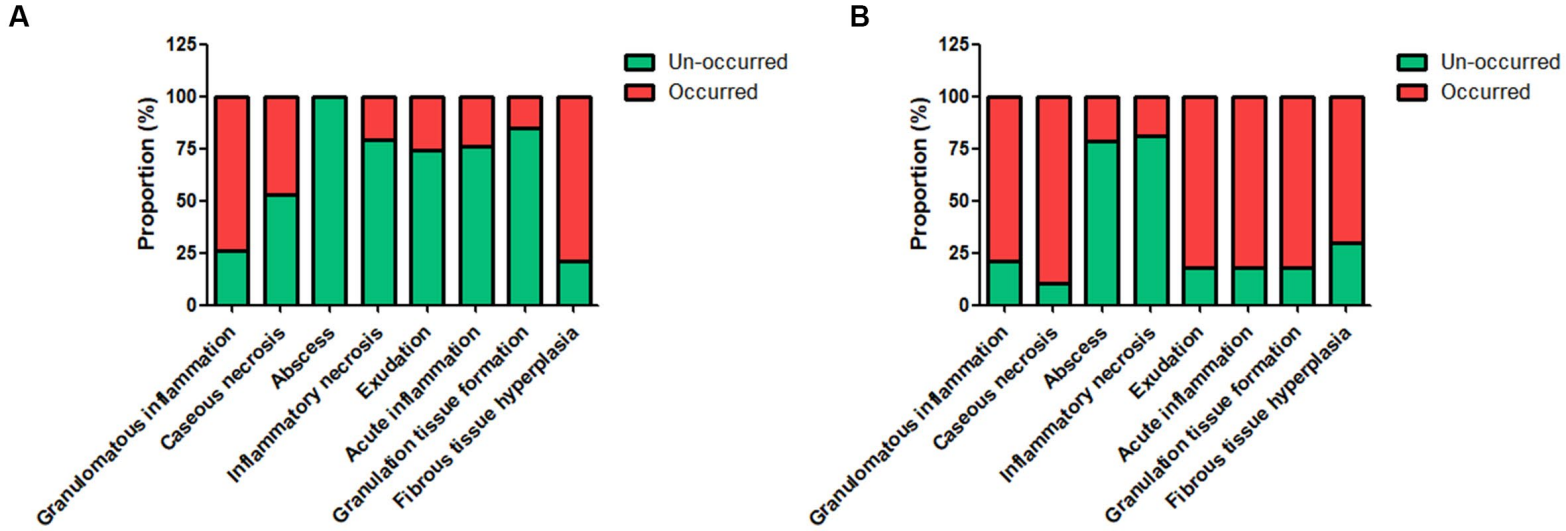
The proportion of different pathological features in the control tuberculosis group [*n* = 207, **(A)**] and spinal tuberculosis group [*n* = 168, **(B)**]. The proportions of caseous necrosis, acute inflammation, abscess, exudation, and granulation tissue formation in the spinal tuberculosis group were all significantly increased relative to the control tuberculosis group.

The control tuberculosis group had 153 cases (73.91%) of typical granulomatous lesions, 47.34% of which were accompanied by typical caseous necrosis. Some cases were complicated with acute inflammation (23.67%), exudation (26.09%), granulation tissue formation (15.46%), inflammatory necrosis (20.77%), and fibrous hyperplasia (78.74%). No abscesses were identified. Typical granulomatous lesions were seen in 133 cases (79.17%) in the spinal tuberculosis group, of which 89.29% were accompanied by typical caseous necrosis. Some cases were associated with acute inflammation (80.95%), exudation (80.95%), granulation tissue formation (80.95%), inflammatory necrosis (19.05%), fibrous tissue hyperplasia (70.24%), and abscess (21.43%). Statistical analysis showed significant differences in the proportion of caseous necrosis, acute inflammation, abscesses, exudation, and granulation tissue formation between the two groups. Acid-fast staining showed 147 cases in the control tuberculosis group and 122 cases in the spinal tuberculosis group were acid-positive, with no statistically significant differences between the two groups (Tables 1, 2 and Figures 3–5).

**Table 1:** Comparison of clinical data and important pathological features.

	Control tuberculosis (*n* = 207)	Spinal tuberculosis (*n* = 168)	*p* value
Gender (Male)	56.52% (*n* = 117)	54.76% (*n* = 92)	0.733
Age (year)	43.86 ± 1.21	43.48 ± 1.35	0.833
TB Type (MTB)	100% (*n* = 207)	100% (*n* = 168)	/
*Methods*			
Acid-fast (+)	71.01% (*n* = 147)	72.62% (*n* = 122)	0.732
PCR (+)	100% (*n* = 207)	100% (*n* = 168)	/
*Pathology*			
Granulomatous inflammation	73.91% (*n* = 153)	79.17% (*n* = 133)	0.194
Caseous necrosis	47.34% (*n* = 98)	89.29% (*n* = 150)	<0.001
Abscess	0% (*n* = 0)	21.43% (*n* = 36)	<0.001
Inflammatory necrosis	20.77% (*n* = 43)	19.05% (*n* = 32)	0.678
Exudation	26.09% (*n* = 54)	80.95% (*n* = 136)	<0.001
Acute inflammation	23.67% (*n* = 49)	80.95% (*n* = 136)	<0.001
Granulation tissue formation	15.46% (*n* = 32)	80.95% (*n* = 136)	<0.001
Fibrous tissue hyperplasia	78.74% (*n* = 163)	70.24% (*n* = 118)	0.059

**Table 2:** pathological features of spinal tuberculosis at different sites.

Spinal tuberculosis	Cervical	Thoracic	Lumbar	Thoracolumbar	Lumbosacral	Total
Number of patients	7	64	82	5	10	168
Granulomatous inflammation	5	53	64	3	8	133
Caseous necrosis	7	55	74	5	9	150
Abscess	0	20	14	0	2	36
Inflammatory necrosis	0	18	13	0	1	32
Exudation	5	49	71	4	7	136
Acute inflammation	4	55	67	3	7	136
Granulation tissue formation	4	53	66	5	8	136
Fibrous tissue hyperplasia	5	46	56	3	8	118

### 3.3. Overall drug resistance

In the control tuberculosis group, susceptibility to all four anti-tuberculosis drugs was observed in 14 samples. Monoresistance was seen in 42.51% of cases, with INH resistance being the most prevalent at 73.86%. Polyresistance was detected in 42.51% of cases, with the highest rate of resistance being to INH + STR, accounting for 41.70%. Multidrug resistance was found in 20.77% of cases, with the highest resistance rate observed for the RFP + INH + STR combination, at 46.51%.

In the spinal tuberculosis group, 16 samples were found to be sensitive to all four anti-tuberculosis drugs. The rate of monoresistance was 41.07%, with INH resistance being the most common, observed in 73.91% of cases. Polyresistance was detected in 26.19% of cases, with the highest rate of resistance being to the RFP + EMB combination, accounting for 29.55%. The multidrug resistance rate was 22.62%, with the highest resistance rate seen for the RFP + INH + STR + EMB combination, at 42.11%.

Statistical analysis revealed that the rates of resistance to RFP + INH + STR and INH + EMB were lower in the spinal tuberculosis group compared to the control tuberculosis group. Conversely, the rates of resistance to RFP + INH + EMB and RFP + EMB were higher in the spinal tuberculosis group. The detailed data is presented in Table 3.

**Table 3:** Comparison of overall drug resistance between the spinal tuberculosis group and the control tuberculosis group.

Drug resistance type	Control tuberculosis group (*n* = 207)	Spinal tuberculosis group (*n* = 168)	*χ*^2^ values	*p* value
*Monoresistance*	88 (42.51)	69 (41.07)	3.704	0.068
RFP resistant	20 (9.66)	13 (7.74)	0.428	0.585
INH resistant	65 (31.40)	51 (30.36)	0.047	0.911
STR resistant	2 (0.97)	3 (1.79)	0.473	0.660
EMB resistant	1 (0.48)	2 (1.19)	0.585	0.585
*Multidrug resistance*	60 (28.99)	44 (26.19)	0.361	0.564
Resistance to RFP + INH	9 (4.35)	6 (3.57)	0.146	0.795
Resistance to RFP + INH + STR	20 (9.66)	6 (3.57)	5.331	0.024
Resistance to RFP + INH + EMB	0 (0.00)	10 (5.95)	12.659	0.000
Resistance to INH + STR + EMB	14 (6.76)	16 (9.52)	0.960	0.345
*Polyresistance*	43 (20.77)	38 (22.62)	0.187	0.706
Resistance to RFP + STR	1 (0.48)	5 (2.98)	3.661	0.094
Resistance to RFP + EMB	5 (2.42)	13 (7.74)	5.749	0.026
Resistance to RFP + STR + EMB	6 (2.90)	10 (5.95)	2.117	0.199
Resistance to INH + STR	25 (12.08)	12 (7.14)	2.539	0.120
Resistance to INH + EMB	20 (9.66)	0 (0.00)	17.146	0.00
Resistance to INH + STR + EMB	3 (1.45)	4 (2.38)	0.439	0.705
Resistance to STR + EMB	0 (0.00)	0 (0.00)	0.00	0.00
*Fully sensitive*	14 (6.76)	16 (9.52)	0.96	0.345
*Any drug resistance*	197 (95.17)	152 (90.48)	3.165	0.101

RFP, rifampicin; INH, isoniazid; STR, streptomycin; EMB, ethambutol. The value outside the parentheses represents “number of patients,” while the value inside the parentheses represents “proportion (%)”.

### 3.4. Drug resistance gene mutation

The control tuberculosis group had 22 mutations in the rpoB gene, of which the 516 codons had the most mutations in D516V (D: aspartic acid →V: valine). Among these mutations, 37 samples showed mutations in one site of a single codon, and 15 samples showed mutations in two sites of a single codon at the same time. katG and inhA mutations were of these types, mainly 315 M mutations, accounting for 57.97%. RpsL mutations were mainly in the form of 88 M mutation, and the EmbB mutations was the 306 M2 mutation.

The spinal tuberculosis group had 22 mutations in the rpoB gene, of which the 516 codons had the most mutations in D516V (D: aspartic acid →V: valine). Among these mutations, 24 samples showed mutations in one site of a single codon, and 14 samples showed in two sites of a single codon at the same time. There were 3 types of katG and inhA mutations, mainly 315 M mutations, accounting for 52.38%. The main form of the RpsL mutations was the 88 M mutation, and the EmbB mutations was the 306 M2 mutation.

In addition, we analyzed their genetic mutations. In the control tuberculosis group, the mutation rate of the D516V + D516G + H526Y + H526D site was 2.90%, but no mutation occurred at this site in the spinal tuberculosis group. The mutation rate of D516V + D516G + H526Y + H526D + S531L + S531W in the spinal tuberculosis group was 3.57%, which was significantly higher than that in the control tuberculosis group. The mutation rate of the EMB306 M2 + 306 M3 site was 2.38% in the spinal tuberculosis group, while no mutation was found at this site in the control tuberculosis group. Specific data was shown in Table 4.

**Table 4:** Comparison of drug-resistant mutation sites between the control tuberculosis group and the spinal tuberculosis group.

Mutational site	Control tuberculosis group (*n* = 207)	Spinal tuberculosis group (*n* = 168)	*χ*^2^ values	*p*-value
*RFP*				
*D516V*	32 (15.46)	20 (11.9)	0.981	0.369
*D516G*	3 (1.45)	0 (0.00)	2.454	0.256
*H526Y*	0 (0.00)	1 (0.60)	1.235	0.448
*S531L*	2 (0.97)	3 (1.97)	0.473	0.660
*D516V + D516G*	13 (6.28)	13 (7.74)	0.305	0.684
*D516V + H526Y*	1 (0.48)	2 (1.19)	0.585	0.589
*D516V + H526D*	6 (2.90)	3 (1.79)	0.49	0.737
*D516V + S531L*	0 (0.00)	0 (0.00)	0.00	0.000
*D516V + D516G + H526D*	8 (3.86)	7 (4.17)	0.022	1.000
*D516V + D516G + S531L*	0 (0.00)	1 (0.60)	1.235	0.448
*D516V + D516G + H526Y + H526D*	6 (2.90)	0 (0.00)	4.949	0.035
*D516V + D516G + H526Y + H526D + S531L*	5 (2.42)	3 (1.79)	0.176	0.736
*D516V + D516G + H526Y + H526D + S531L + S531W*	1 (0.48)	6 (3.57)	4.829	0.048
*D516V + D516G + H526D + S531L*	1 (0.48)	4 (2.38)	2.539	0.178
*D516V + H526Y + H526D*	1 (0.48)	1 (0.60)	0.022	1.000
*D516V + H526Y + H526D + S531L*	0 (0.00)	1 (0.60)	1.235	0.448
*D516V + H526D + S531L*	2 (0.97)	1 (0.60)	0.161	1.000
*D516G + H526Y*	0 (0.00)	1 (0.60)	1.235	0.448
*D516G + H526D*	1 (0.48)	1 (0.60)	0.022	1.000
*H526Y + H526D*	2 (0.97)	1 (0.60)	0.161	1.000
*H526Y + H526D + S531L*	0 (0.00)	1 (0.60)	1.235	0.448
*H526D + S531L*	1 (0.48)	0 (0.00)	0.814	1.000
*INH*				
*315 M*	120 (57.97)	88 (52.38)	1.618	0.211
*-15 M*	6 (2.90)	6 (3.57)	0.136	0.773
*-15 M + 315 M*	25 (12.08)	24 (14.29)	0.398	0.541
*STR*				
*43 M*	15 (7.25)	10 (5.95)	0.617	0.282
*88 M*	41 (19.81)	29 (17.26)	0.396	0.595
*43 M + 88 M*	14 (6.76)	16 (9.52)	0.96	0.345
*EMB*				
*306 M1*	2 (0.97)	4 (2.38))	1.179	0.414
*306 M2*	36 (17.39)	36 (21.43)	0.974	0.357
*306 M1 + 306 M2*	11 (5.31)	12 (7.14)	0.539	0.520
*306 M2 + 306 M3*	0 (0.00)	4 (2.38)	4.982	0.039
*306 M1 + 306 M2 + 306 M3*	2 (0.97)	3 (1.79)	0.473	0.660

RFP, rifampicin; INH, isoniazid; STR, streptomycin; EMB, ethambutol; D, Aspartate; V, Valine, G, Glycine; H: Histidine, Y: tyrosine, S: serine, L: leucine, W: tryptophan; The value outside the parentheses represents “number of patients,” while the value inside the parentheses represents “proportion (%)”.

## 4. Discussion

Differences in the epidemiological characteristics, pathogenesis, and pathological features between lung tuberculosis and spinal tuberculosis have been noted (Pandita et al., 2020). Lung tuberculosis is the most common type of tuberculosis and is transmitted mainly by airborne droplets (Torrelles and Schlesinger, 2017). It causes lung infection, leading to symptoms such as cough, sputum, and chest pain, which can be life-threatening in severe cases (Ravimohan et al., 2018). Spinal tuberculosis is a rare but serious form of tuberculosis, usually caused by the spread of *Mycobacterium tuberculosis* to the spine through the bloodstream or lymphatic system (Kumar, 2016). Spinal tuberculosis often presents with symptoms of spinal pain, deformity, and nerve root compression, which may cause spinal cord injury and paralysis in severe cases (Kumar, 2016). Standard anti-tuberculosis therapy remains the mainstay of treatment for lung tuberculosis and spinal tuberculosis, and effective for most of the patients (Singh et al., 2014). For the patients who are not promptly or effectively treated, lung tuberculosis can progress to spinal tuberculosis. Therefore, the timely diagnosis and treatment of lung tuberculosis is important for preventing spinal tuberculosis.

Spinal tuberculosis exhibits unique pathological features. In the early stage, *Mycobacterium tuberculosis* invades the vertebral body and the intervertebral disc, resulting in caseous necrosis (Liu et al., 2019). Caseous necrosis is a special type of coagulated necrosis, which is characterized by unstructured granular red staining substance. As caseous necrosis progresses, the vertebral body gradually collapses, leading to deformity (Mittal et al., 2016); This deformity can cause abnormal spinal curvature and overall spinal deformity. In the late stage of spinal tuberculosis, the caseous material can be reabsorbed, leading to vertebral body hollowing (Izawa, 2015). Simultaneously, infectious inflammation can impact the surrounding bone tissue, potentially leading to dense stomatitis (Izawa, 2015); When the vertebral body becomes deformed, collapses, and hollows out, it may result in nerve root subluxation and compression (Chen et al., 2022); These complications can cause weakness in the lower extremities, paresthesia, urinary dysfunction, and other serious issues. Study showed that the most common pathologic characteristics were caseous necrosis, multinuclear giant cells, and granulomatous inflammation (Li et al., 2020). Our study revealed that 89.19% of patients with spinal tuberculosis developed typical caseous necrosis, a significantly higher rate compared to the control tuberculosis group. This high incidence in the spinal tuberculosis group may be attributed to the unique anatomy and tissue structure around the spine, circulation deprivation, and poor necrotic material drainage. Additionally, the spinal tuberculosis group showed significantly higher occurrences of acute inflammation, abscesses, exudates, and granulation tissue formation than the control tuberculosis group. These unique pathological features significantly increase the difficulty of diagnosis and treatment of spinal tuberculosis.

Classical resistance testing involves the isolation and culture of *mycobacterium tuberculosis* and therefore requires high-level biosafety laboratory and professional operators, which is difficult for pathology departments to achieve. The emergence of drug resistance in *Mycobacterium tuberculosis* is primarily driven by genetic mutations, and identifying these mutations can help inform clinical treatment (Phelan et al., 2016). Resistance to isoniazid typically involves mutations in the katG and inhA promoter regions, particularly at the katG315 site (Seifert et al., 2015). Mutations in the rpoB gene are one of the main mechanisms leading to rifampin resistance, and these mutations occur mainly in the rifampin resistance determining region (RRDR), accounting for more than 95% of all rpoB resistance-associated mutations (Zaw et al., 2018). Mutations in embB and its operon embCAB are closely linked to resistance to ethambutol, and mutations occur most frequently in the ethambutol resistance determining region (ERDR) (Sun et al., 2018). Our results showed that the spinal tuberculosis group differed from the control tuberculosis group in terms of drug resistance and drug resistance gene mutations. Notably, higher proportions of RFP + INH + EMB and RFP + EMB resistance were observed in the spinal tuberculosis group. The reason for this difference may be that the blood circulation in the spinal tuberculosis site is relatively poor, anti-tuberculosis drugs cannot reach effective killing concentrations in caseous necrotic tissue, and residual *mycobacterium tuberculosis* may undergo drug resistance changes. Thus, it is essential to consider these distinct drug resistance profiles when selecting antituberculosis medications. Personalized treatment plans should be developed, considering drug sensitivity test results and individual patient conditions. If treatment efficacy is lacking, immediate measures such as a spinal tap or biopsy should be considered.

Our findings may help in the diagnosis and treatment of spinal tuberculosis. Some spinal tuberculosis showed high metabolic value on PET-CT examination, which was easily misdiagnosed as spinal malignancy or metastatic cancer. It may be related to high glucose metabolism around the focal of tuberculosis. The pathological features of spinal tuberculosis were more complex, and the incidence of caseous necrosis, acute inflammation, abscess, exudation, and granulation tissue formation were significantly higher than those of pulmonary tuberculosis. In the pathological diagnosis, we should rule out the possibility of spinal tuberculosis, even if it only shows acute inflammation, abscision, expulsion, or granulomatous tissue formation. We found some differences in both drug resistance and drug resistance gene mutations, suggesting that there may be some differences in treatment options for spinal tuberculosis and other tuberculosis. Before treating spinal tuberculosis, we should test for drug resistance whenever possible, if conditions permit. However, this study does have some limitations. The relatively small number of cervical tuberculosis cases may not fully represent the disease’s pathological characteristics. Additionally, the genetic testing failed to detect resistance to second-line anti-tuberculosis drugs, which detracts from its clinical usefulness. In our subsequent studies, we aim to include more cases and expand the scope of testing for drug resistance genes.

In conclusion, the proportion of caseous necrosis, acute inflammation, abscess, exudation, and granulation tissue formation increased significantly in the spinal tuberculosis group, and the drug resistance characteristics and drug resistance gene mutations were also different from the control tuberculosis group. This study provides important data to support the diagnosis and treatment of spinal tuberculosis.

## Data availability statement

The original contributions presented in the study are included in the article/Supplementary material, further inquiries can be directed to the corresponding authors.

## Ethics statement

This study was approved by the Ethics Committee of the Chinese PLA General Hospital and was performed in accordance with the principles of the Declaration of Helsinki.

## Author contributions

WC and FW participated in research design. RW, YL, and DL participated in the collection of TB cases. SL and YL performed the experiments. RW performed the data analysis. RW and SL participated in the writing of the manuscript. FW and WC performed visualization. RW, HZ, and SL edited language. All authors contributed to the article and approved the submitted version.

## Funding

This work was supported by the National Natural Science Foundation of China (nos: 31800814, 32271411).

## Conflict of interest

The authors declare that the research was conducted in the absence of any commercial or financial relationships that could be construed as a potential conflict of interest.

## Publisher’s note

All claims expressed in this article are solely those of the authors and do not necessarily represent those of their affiliated organizations, or those of the publisher, the editors and the reviewers. Any product that may be evaluated in this article, or claim that may be made by its manufacturer, is not guaranteed or endorsed by the publisher.
