# Supplementary material for: A high proportion of caseous necrosis, abscess, and granulation tissue formation in spinal tuberculosis
Source: Front Microbiol. 2023 Aug 14;14:1230572. doi: 10.3389/fmicb.2023.1230572 (PMC10461047; doi:10.3389/fmicb.2023.1230572)
Supplement: Supplementary file 1 [file Data_Sheet_1.docx]

**A high proportion of caseous necrosis, abscess, and granulation tissue formation in spinal tuberculosis**

**Runrui Wu ^a^, ShanShan Li ^a^,Yadong Liu , Hong Zhang,Dongxu Liu ,Yuejiao Liu**

**Wen Chen ^a^, Fenghua Wang ^a^**

a. Department of Pathology, The 8th Medical Center, Chinese PLA General Hospital, Beijing 100091, China

Runrui Wu and Shanshan Li contributed equally to this work.

Corresponding author: Fenghua Wang, E-mail: 13810915901@163.com; Wen Chen, E-mail: dr.chen20160224@foxmail.com

Figure S1. PET-CT (A) and CT (B) examinations for pulmonary tuberculosis.
